# Common gastrointestinal diseases and chronic obstructive pulmonary disease risk: a bidirectional Mendelian randomization analysis

**DOI:** 10.3389/fgene.2023.1256833

**Published:** 2023-11-16

**Authors:** Zixiong Shen, Binxu Qiu, Lanlan Chen, Yiyuan Zhang

**Affiliations:** ^1^ Department of Thoracic Surgery, The First Hospital of Jilin University, Changchun, China; ^2^ Department of Gastrointestinal Surgery, The First Hospital of Jilin University, Changchun, China; ^3^ Hepatobiliary and Pancreatic Surgery, The First Hospital of Jilin University, Changchun, China

**Keywords:** chronic obstructive pulmonary disease, gastrointestinal diseases, gastroesophageal reflux disease, peptic ulcer disease, irritable bowel syndrome, constipation, Mendelian randomization

## Abstract

**Background:** Observational studies suggest an association between gastrointestinal diseases and chronic obstructive pulmonary disease (COPD), but the causal relationship remains unclear.

**Methods:** We conducted bidirectional Mendelian randomization (MR) analysis using summary data from genome-wide association study (GWAS) to explore the causal relationship between common gastrointestinal diseases and COPD. Gastrointestinal diseases included gastroesophageal reflux disease (GERD), peptic ulcer disease (PUD), irritable bowel syndrome (IBS), Crohn’s disease (CD), ulcerative colitis (UC), functional dyspepsia (FD), non-infectious gastroenteritis (NGE), and constipation (CP). Significant MR analysis results were replicated in the COPD validation cohort.

**Results:** Bidirectional MR analysis supported a bidirectional causal relationship between GERD and COPD, and COPD was also found to increase the risk of IBS and CP. Our study also provided evidence for a bidirectional causal relationship between PUD and COPD, although the strength of evidence may be insufficient. Furthermore, we provided evidence that there is no causal association between CD, UC, FD, NGE, and COPD.

**Conclusion:** This study offers some evidence to clarify the causal relationship between common gastrointestinal diseases and COPD. Further research is needed to understand the underlying mechanisms of these associations.

## Introduction

Chronic obstructive pulmonary disease (COPD) is one of the most common chronic respiratory diseases, with a forced expiratory volume in one second to forced vital capacity ratio (FEV1/FVC ratio) less than 70% after bronchodilator is the gold standard for COPD diagnosis (Halpin et al., 2021). COPD affects 544.9 million people globally and is the third leading cause of death in the past decades, accounting for 7% of all deaths (GBD Chronic Respiratory Disease Collaborators, 2020). Observational studies have indicated an association between gastrointestinal diseases and COPD (Wang et al., 2023), but the precise causal relationship has not been confirmed.

Gastroesophageal reflux disease (GERD) involves the reflux of stomach contents into the esophagus, causing various uncomfortable symptoms. Approximately 20% of adults in the Western world have GERD (Maret-Ouda et al., 2020a). Observational studies have found that GERD is one of the most common complications of COPD (Zou et al., 2022), and the risk of worsening COPD in GERD patients is doubled (Rascon-Aguilar et al., 2006). GERD and COPD appear to have a mutually reinforcing effect, not merely a shared susceptibility to certain environmental factors. Peptic ulcer disease (PUD) primarily includes open ulcers in the gastric mucosa and upper small intestine, and it can also occur in the lower esophagus, small intestine, gastrojejunal anastomosis, etc (Lanas and Chan, 2017). The estimated lifetime prevalence of peptic ulcer disease is approximately 5%–10% (Lanas and Chan, 2017). Observational studies have found that the risk of PUD is increased by 17%–24% in COPD patients (Schneider et al., 2010; Huang et al., 2017). Irritable bowel syndrome (IBS) is a functional gastrointestinal disorder characterized by symptoms related to changes in stool form or frequency, and it affects approximately 5%–10% of the global population (Ford et al., 2020a). A cohort study involving 14,021 IBS patients found a significant increase in the risk of COPD in IBS patients (*p* < 0.0001) (Lai et al., 2020), with a hazard ratio (HR) of 1.512. Another study also found an increased risk of IBS in COPD patients (HR = 1.55, *p* < 0.001) (Chiu et al., 2022). Inflammatory bowel disease (IBD) includes Crohn’s disease (CD) and ulcerative colitis (UC) and is a chronic, recurrent gastrointestinal disease (Wehkamp et al., 2016). Approximately 1 in 198 individuals has UC, and 1 in 310 individuals has CD (Wehkamp et al., 2016). IBD has numerous extraintestinal manifestations, including pulmonary manifestations (Rogler et al., 2021). A retrospective cohort study in Canada indicated a 55% higher incidence of CD and a 30% higher incidence of UC in COPD patients (Brassard et al., 2015). There is also evidence linking severe IBD progression to pulmonary inflammation and pathology (Vutcovici et al., 2016; Raftery et al., 2023). Functional dyspepsia (FD) encompasses a range of symptoms involving the gastroduodenal region, including upper abdominal pain or burning sensations and postprandial fullness or early satiety (Ford et al., 2020b). FD affects approximately 18% of the general population (Ford et al., 2020b). Observational studies have shown a significant increase in the risk of FD in COPD patients (HR = 1.34, *p* < 0.003) (Chiu et al., 2022). Non-infectious gastroenteritis (NGE) is a group of diseases, including gastritis, duodenitis, and esophagitis, typically not caused by infections (Mathews et al., 2022). Globally, more than half of the population may suffer from chronic gastrointestinal inflammation (Sipponen and Maaroos, 2015), and COPD patients appear to have a higher prevalence of NGE (Fedorova et al., 2003). Constipation (CP) is another common functional gastrointestinal disorder, affecting approximately 16% of adults (Bharucha and Wald, 2019). Previous studies have shown an increased risk of CP in COPD patients (Sun et al., 2013; Gursoy Coskun et al., 2021). The aforementioned observational studies suggest a complex interplay between gastrointestinal diseases and COPD, which is also referred to as the “gut-lung axis” (Gokulan et al., 2022).

There seems to be a delicate relationship between gastrointestinal diseases and COPD. Understanding the potential causal relationships can aid in better disease management, with the aim of improving patient prognosis. However, there are currently no appropriate randomized controlled trials (RCTs) to elucidate these causal associations. Designing RCTs for these gastrointestinal diseases and COPD is impractical due to confounding factors such as smoking, obesity, and asthma. In the absence of feasible RCTs, Mendelian randomization (MR) is a reliable causal inference method (Skrivankova et al., 2021). MR uses genetic variations as instrumental variables (IVs) and provides evidence regarding the causal relationship between modifiable exposure factors and diseases (Skrivankova et al., 2021). MR is less susceptible to confounding and reverse causation and is effective in reducing financial and human resource costs (Davies et al., 2018). To provide more evidence to clarify the causal relationship between gastrointestinal diseases (GERD, PUD, IBS, CD, UC, FD, NGE, and CP) and COPD, this study explored the potential causal effects using bidirectional MR.

## Materials and methods

### Study design

**FIGURE 1:**
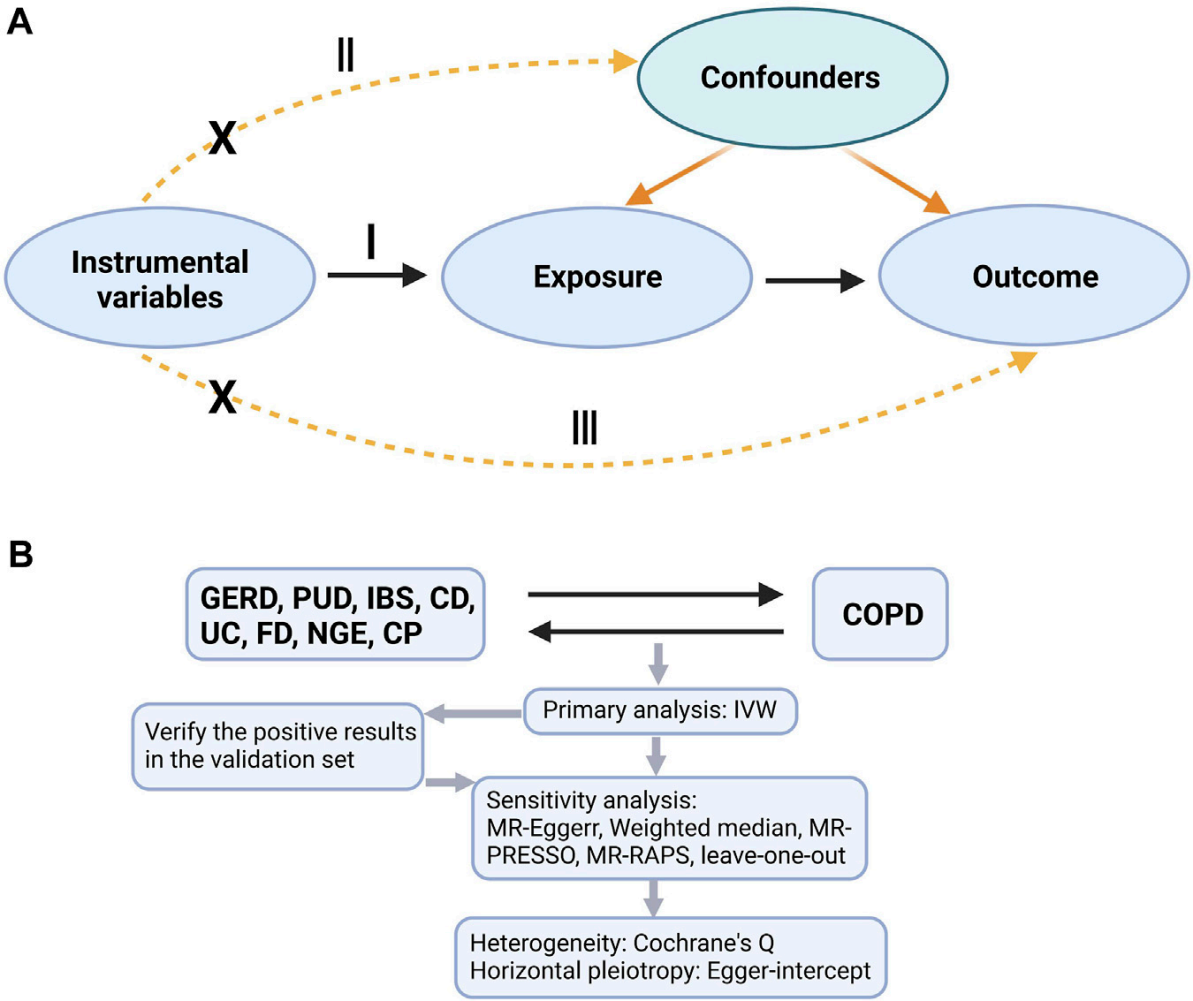
A is a bidirectional acyclic graph. B is an overview of our study. I: assumption I, II: assumption II; III: assumption III; GERD, gastroesophageal reflux disease; PUD, peptic ulcer disease; IBS, irritable bowel syndrome; CD, Crohn’s disease; UC, ulcerative colitis; FD, functional dyspepsia; NGE, noninfectious gastroenteritis; CP, constipation; COPD, chronic obstructive pulmonary disease; IVW, inverse variance weighting; MR-Egger, MR-Egger regression; MR-PRESSO, Mendelian Randomization Pleiotropy RESidual Sum and Outlier (MR-PRESSO) test; MR-RAPS, Mendelian Randomization Robust Adjusted Profile Score.

An overview of the research design is shown in Figure 1. Our research is based on three main assumptions of MR research (Haycock et al., 2016). I: The instrumental variable is related to exposure; II: The instrumental variable is not related to any known or unknown confounding factors that can mediate from exposure to outcome; III: The outcome is associated with the genetic instrument only through the effect of the exposure.

### GWAS summary data source

The summary data for GERD is derived from a genome-wide association study (GWAS) meta-analysis conducted by Ong et al. (2022), which includes 129,080 cases of European ancestry and 473,524 European ancestry controls. The summary data for PUD is obtained from a GWAS meta-analysis by Wu et al. (2021), comprising 16,666 European ancestry cases and 439,661 European ancestry controls. The GWAS summary data for IBS is sourced from Eijsbouts et al.'s report (Eijsbouts et al., 2021), encompassing 53,400 cases of European ancestry and 433,201 European ancestry controls. The summary data for CD and UC are obtained from Lange et al.'s report (de Lange et al., 2017). CD includes 12,194 cases of mixed ancestry and 28,072 mixed ancestry controls, while UC includes 12,366 cases of mixed ancestry and 33,609 mixed ancestry controls. The summary data for FD is sourced from FinnGen (https://www.finngen.fi/en) (Kurki et al., 2023), including 8,875 European cases and 320,387 European controls. The data for NGE is obtained from Jiang et al.’s analysis of the UK Biobank data (Jiang et al., 2021), comprising 11,373 European cases and 444,975 European controls. The summary data for CP is sourced from a trans-ethnic GWAS meta-analysis conducted by Sakaue et al. (2021), which includes 16,299 cases of European and East Asian mixed ancestry and 571,953 European and East Asian mixed ancestry controls. The summary data for COPD (discovery) is obtained from Sakaue et al.'s trans-ethnic GWAS meta-analysis (Sakaue et al., 2021), which includes 17,574 cases of European and East Asian mixed ancestry and 617,598 European and East Asian mixed ancestry controls. COPD (validation) data is from FinnGen, comprising 18,266 European ancestry cases and 311,286 European ancestry controls. Ethical approval for each of the summary datasets is available in the original studies, and these data can be used without restrictions. Table 1 provides a brief overview of the summary data.

**TABLE 1:** A brief description of each GWAS summary statistics.

Combination	Ancestry	Sample size of exposure	PMID	Group	NSNP	R2 (%)	F
GERD-COPD	European	129,080 cases, 473,524 controls	34,187,846	Discovery	40	1.66	253.66
				Validation	39	1.61	252.49
PUD-COPD	European	16,666 cases, 439,661 controls	33,608,531	Discovery	36	5.72	768.69
				Validation	34	5.46	774.85
IBS-COPD	European	53,400 cases, 433,201 controls	34,741,163	Discovery	63	3.66	293.80
CD-COPD	Mixed	12,194 cases, 28,072 controls	28,067,908	Discovery	50	55.40	999.21
UC-COPD	Mixed	12,366 cases, 33,609 controls	28,067,908	Discovery	42	39.55	715.50
FD-COPD	European	8,875 cases, 320,387 controls	—	Discovery	14	3.62	884.56
NGE-COPD	European	11,373 cases, 444,975 controls	34,737,426	Discovery	19	3.78	944.44
CP-COPD	Mixed	15,902 European ancestry cases, 395,721 European ancestry controls, 397 East Asian ancestry cases, 176,232 East Asian ancestry controls	34,594,039	Discovery	21	4.04	1180.00
COPD-GERD	Mixed	13,530 European cases, 454,945 European controls, 4,017 East Asian cases, 162,653 East Asian controls	34,594,039	Discovery	31	4.59	986.16
	European	18,266 European cases, 311,286 European controls	—	Validation	31	4.50	501.09
COPD-PUD	Mixed	13,530 European cases, 454,945 European controls, 4,017 East Asian cases, 162,653 East Asian controls	34,594,039	Discovery	53	8.44	1104.63
	European	18,266 European cases, 311,286 European controls	—	Validation	70	10.42	547.53
COPD-IBS	Mixed	13,530 European cases, 454,945 European controls, 4,017 East Asian cases, 162,653 East Asian controls	34,594,039	Discovery	60	12.37	1494.22
COPD-IBS	European	18,266 European cases, 311,286 European controls	—	Validation	75	11.18	552.91
COPD-CD	Mixed	13,530 European cases, 454,945 European controls, 4,017 East Asian cases, 162,653 East Asian controls	34,594,039	Discovery	55	11.25	1464.44
COPD-UC	Mixed	13,530 European cases, 454,945 European controls, 4,017 East Asian cases, 162,653 East Asian controls	34,594,039	Discovery	55	11.25	1464.44
COPD-FD	Mixed	13,530 European cases, 454,945 European controls, 4,017 East Asian cases, 162,653 East Asian controls	34,594,039	Discovery	58	11.80	1464.72
COPD-NGE	Mixed	13,530 European cases, 454,945 European controls, 4,017 East Asian cases, 162,653 East Asian controls	34,594,039	Discovery	57	9.34	1148.39
COPD-CP	Mixed	13,530 European cases, 454,945 European controls, 4,017 East Asian cases, 162,653 East Asian controls	34,594,039	Discovery	61	12.74	1520.40
	European	18,266 European cases, 311,286 European controls	—	Validation	76	10.99	535.02

NSNP, Number of SNPs; R2, exposure variance explained by all IVs; F, F-statistic; GERD, gastroesophageal reflux disease; PUD, peptic ulcer disease; IBS, irritable bowel syndrome; CD, Crohn’s disease; UC, ulcerative colitis; FD, functional dyspepsia; NGE, noninfectious gastroenteritis; CP, constipation; COPD, chronic obstructive pulmonary disease.

### Instrumental variables

We extracted SNPs strongly associated with GERD, CD, and UC using a threshold of *p* < 5.0E-08. To ensure an adequate number of SNPs, we used a threshold of *p* < 1.0E-05 to extract SNPs for PUD, IBS, FD, NGE, CP, and both the discovery and validation sets of COPD. We calculated the F-statistic for each SNP as well as the overall F-statistic for all SNPs. The F-statistic for an individual SNP was calculated using the following formula (Codd et al., 2013): $F=\frac{{\text{beta}}^{2}}{{\text{se}}^{2}}$ , where “beta” is the effect size of the SNP on the exposure, and “se” is the standard error corresponding to “beta.” The overall F-statistic was calculated using the following formula (Codd et al., 2013): $F=\frac{N-K-1}{K}$ × $\frac{R^{2}}{1-R^{2}}$ , $R^{2}=2\times \text{eaf}\times \left(1\;-\text{eaf}\right)\times {\text{beta}}^{2}$ , where N is the sample size of the exposure, K is the number of IVs, *R* ^2^ is the proportion of exposure variance explained by the SNPs, eaf is the effect allele frequency of the SNP, and beta is the effect size of the SNP on the exposure. An F-statistic greater than 10 indicates a strong association between the SNP and the phenotype (Lawlor et al., 2008). The *R* ^2^ and overall F-statistic for all causal estimates in this study can be found in Table 1, and individual SNP F-statistics are described in Supplementary Table S2.

We applied strict criteria for removing linkage disequilibrium, with a clustering window set at 10,000 kb and an *r* ^2^ threshold set at 0.001. We also removed palindromic SNPs with intermediate allele frequencies (minor allele frequency >0.42). Additionally, based on a threshold of *p* < 1.0E-05, we searched all SNPs in the PhenoScanner database, and SNPs associated with the outcome or potential confounders were excluded.

Previous studies have shown a strong association between asthma and COPD, and both gastrointestinal diseases and asthma are influenced by smoking (Hikichi et al., 2018). Among the instrumental variables (IVs) representing gastrointestinal diseases, SNPs related to smoking, asthma, and lung function will be excluded. Previous research has also indicated an increased risk of gastrointestinal diseases with asthma (Althoff and Sharma, 2023), and both gastrointestinal diseases and COPD are susceptible to the influence of smoking (Berkowitz et al., 2018; Maret-Ouda et al., 2020b). Therefore, when selecting IVs for COPD, SNPs related to smoking and asthma will be excluded. Furthermore, considering the influence of obesity on GERD (Friedenberg et al., 2008), when analyzing the causal effect of COPD on GERD, SNPs related to body mass index will be excluded from the IVs for COPD.

### Statistical analysis

We conducted MR analysis on 8 pairs of causal combinations: GERD-COPD, PUD-COPD, IBS-COPD, CD-COPD, UC-COPD, FD-COPD, NGE-COPD, and CP-COPD, where GERD, PUD, IBS, CD, UC, FD, NGE, and CP were treated as exposures and COPD as the outcome. Subsequently, reverse MR analysis was performed with COPD as the exposure to obtain COPD-GERD, COPD-PUD, COPD-IBS, COPD-CD, COPD-UC, COPD-FD, COPD-NGE, and COPD-CP, totaling 8 reverse causal combinations. Finally, validation analysis was carried out on the positive results using the COPD validation dataset.

In the primary analysis, the Inverse Variance Weighting (IVW) random-effects model was used to estimate the combined causal effects (Bowden et al., 2017). Sensitivity analyses included MR-Egger regression (Bowden et al., 2015), Weighted Median (Bowden et al., 2016a), Mendelian Randomization Pleiotropy RESidual Sum and Outlier (MR-PRESSO) (Verbanck et al., 2018), and Mendelian Randomization Robust Adjusted Profile Score (MR-RAPS) (Zhao et al., 2020). MR-PRESSO was used to detect potential horizontal pleiotropy and, if significant, provide IVW estimates corrected for outliers (Verbanck et al., 2018). In this study, MR-PRESSO was set with a Distribution of 1000 and SignifThreshold of 0.05. Cochrane’s Q and I^2^ statistics were calculated to assess heterogeneity (Bowden et al., 2016b; Bowden et al., 2019), with I^2^>90% indicating robust and reliable results (Bowden et al., 2016b). Furthermore, MR-Egger regression intercept testing was conducted to assess horizontal pleiotropy, with a non-zero intercept suggesting the presence of horizontal pleiotropy (Bowden et al., 2017). Finally, we performed leave-one-out analyses to ensure that our MR results were not unduly influenced by individual SNPs.

MR results were presented as odds ratios (ORs), with estimates primarily used to determine the direction of causality (Burgess and Labrecque, 2018). In the bidirectional MR analysis of gastrointestinal diseases and COPD, causal estimates were conducted in both directions for all 8 combinations. Considering multiple testing contributions, we considered MR results statistically significant when *p* < 0.05/8 = 0.00625 based on the Bonferroni correction, and suggestive significance when 0.00625 < *p* < 0.05. All analyses were performed using the open-source statistical software R (version: 4.2.3). MR analysis was based on “TwoSampleMR (https://github.com/MRCIEU/TwoSampleMR.git),” “mr.raps (https://github.com/qingyuanzhao/mr.raps.git),” and “MR-PRESSO (https://github.com/rondolab/MR-PRESSO.git).” Data visualization was based on “TwoSampleMR” and “forestploter (https://github.com/adayim/forestploter.git).”

## Results

### IVs for gastrointestinal diseases

In MR analyses with gastrointestinal diseases as exposures, preliminary analyses initially identified 80, 41, 63, 89, 58, 20, 24, and 24 IVs for GERD, PUD, IBS, CD, UC, FD, NGE, and CP, respectively. After screening these IVs in the PhenoScanner database, 29 IVs from GERD, 3 IVs from PUD, 8 IVs from IBS, 30 IVs from CD, 10 IVs from UC, and 1 IV from FD were found to have potential pleiotropy or relevance to the outcomes. Harmonization with discovery data for COPD was performed, which involved the removal of palindromic structure SNPs and missing SNPs in the outcomes. Finally, 40, 36, 63, 50, 42, 14, 14, and 21 SNPs remained as IVs for GERD-COPD, PUD-COPD, IBS-COPD, CD-COPD, UC-COPD, FD-COPD, NGE-COPD, and CP-COPD, respectively. MR analysis for GERD-COPD and PUD-COPD yielded positive results, leading us to perform validation analyses using COPD validation data. Following the same IV selection process, 39 and 34 SNPs were selected as IVs for validation analysis in GERD-COPD and PUD-COPD, respectively.

### IVs for COPD

In MR analyses with COPD as the exposure, we initially obtained 81 and 103 SNPs as discovery and validation IVs for COPD, respectively. In the COPD-GERD MR analysis, we excluded 19 SNPs with potential pleiotropy from the COPD discovery IVs and 18 SNPs with potential pleiotropy from the COPD validation IVs based on the PhenoScanner database. In the COPD-PUD, COPD-IBS, COPD-CD, COPD-UC, COPD-FD, COPD-NGE, and COPD-CP MR analyses, 14 SNPs with potential pleiotropy were identified from the COPD discovery IVs, along with 13 SNPs with potential pleiotropy from the COPD validation IVs based on the PhenoScanner database. Harmonization was conducted between COPD discovery data and gastrointestinal disease data, involving the removal of palindromic structure SNPs and missing SNPs in the outcomes. Ultimately, 31, 53, 60, 55, 55, 58, 57, and 61 SNPs remained as IVs for COPD-GERD, COPD-PUD, COPD-IBS, COPD-CD, COPD-UC, COPD-FD, COPD-NGE, and COPD-CP, respectively. Positive MR results were obtained for COPD-GERD, COPD-PUD, COPD-IBS, and COPD-CP, prompting validation analyses using COPD validation data. Following the same IV selection process, 31, 70, 75, and 76 SNPs were chosen as IVs for validation analysis in COPD-GERD, COPD-PUD, COPD-IBS, and COPD-CP, respectively. Supplementary Tables S1, S2 provide basic information on the SNPs that were excluded in this study and those used in the final MR analysis, respectively.

### MR Results and Sensitivity Analysis

**FIGURE 2:**
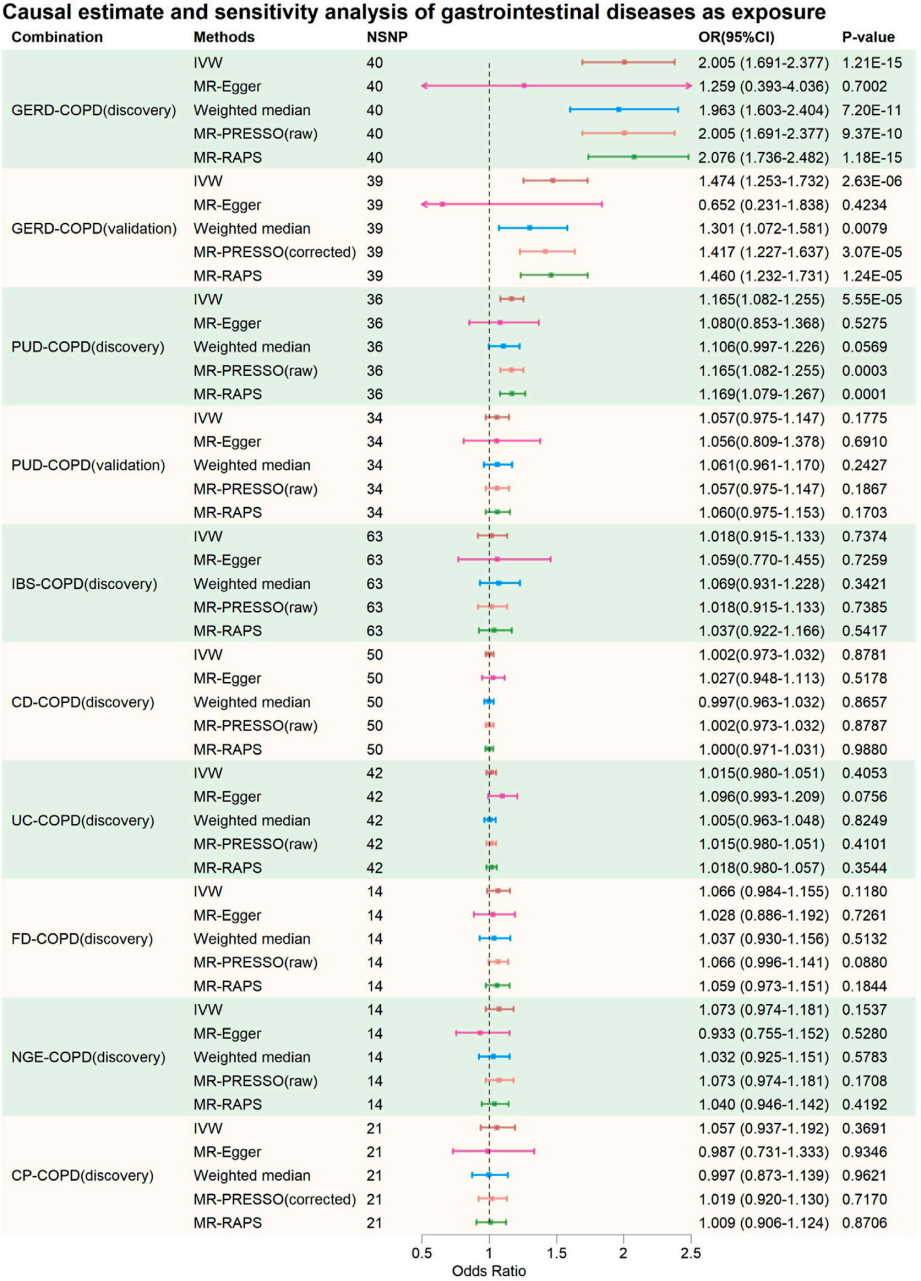
Causal estimate and sensitivity analysis of gastrointestinal diseases as exposure. NSNP, Number of SNPs; OR, odds ratio; 95%LCI, lower limit of 95% confidence interval of OR; 95%UCI, upper limit of 95% confidence interval of OR; *p*-value, *p*-value of OR; IVW, inverse variance weighting; MR-Egger, MR-Egger regression; MR-PRESSO, Mendelian Randomization Pleiotropy RESidual Sum and Outlier (MR-PRESSO) test; MR-RAPS, Mendelian Randomization Robust Adjusted Profile Score; GERD, gastroesophageal reflux disease; PUD, peptic ulcer disease; IBS, irritable bowel syndrome; CD, Crohn’s disease; UC, ulcerative colitis; FD, functional dyspepsia; NGE, noninfectious gastroenteritis; CP, constipation; COPD, chronic obstructive pulmonary disease.

**FIGURE 3:**
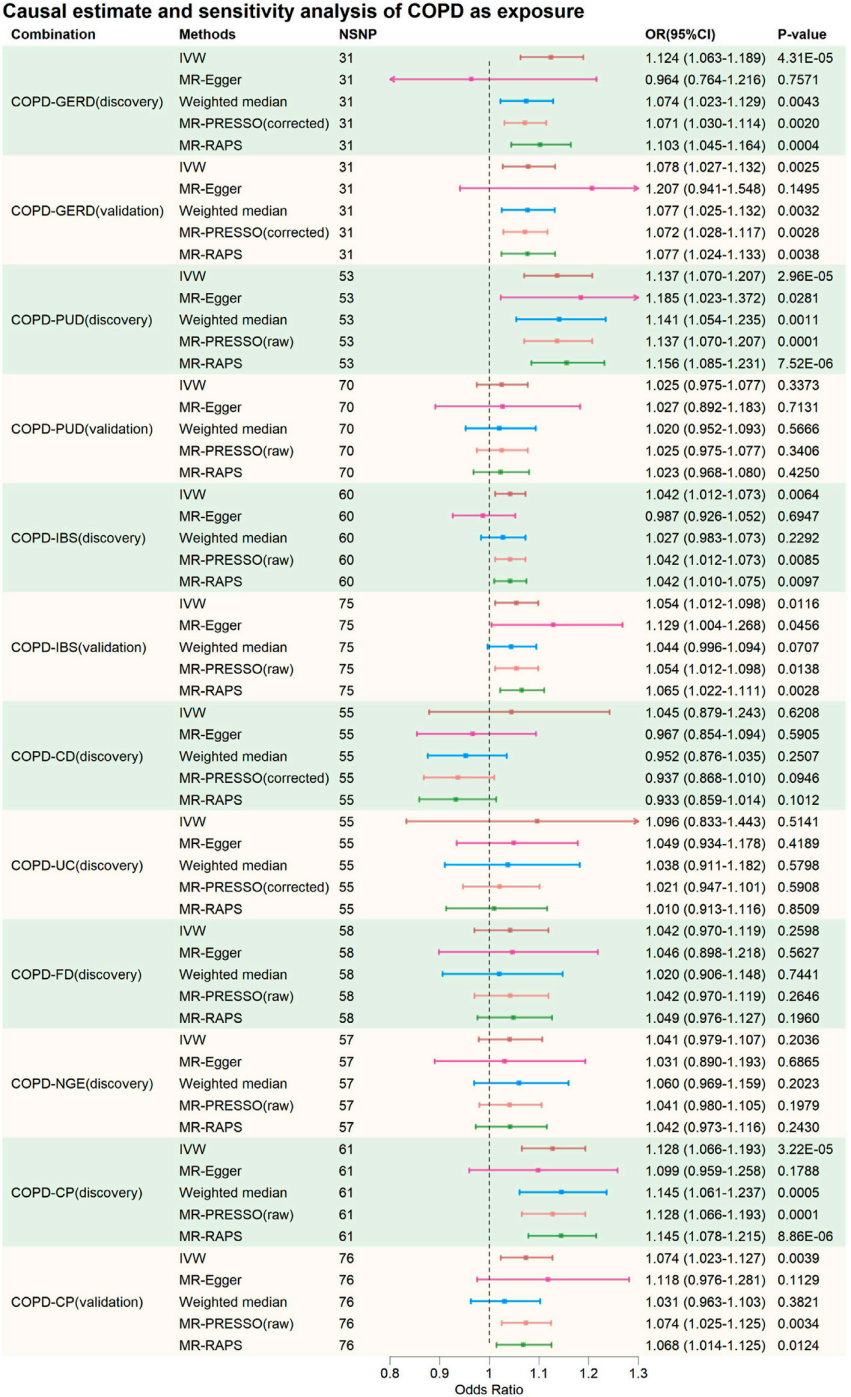
Causal estimate and sensitivity analysis of COPD as exposure. NSNP, Number of SNPs; OR, odds ratio; 95%LCI, lower limit of 95% confidence interval of OR; 95%UCI, upper limit of 95% confidence interval of OR; *p*-value, *p*-value of OR; IVW, inverse variance weighting; MR-Egger, MR-Egger regression; MR-PRESSO, Mendelian Randomization Pleiotropy RESidual Sum and Outlier (MR-PRESSO) test; MR-RAPS, Mendelian Randomization Robust Adjusted Profile Score; GERD, gastroesophageal reflux disease; PUD, peptic ulcer disease; IBS, irritable bowel syndrome; CD, Crohn’s disease; UC, ulcerative colitis; FD, functional dyspepsia; NGE, noninfectious gastroenteritis; CP, constipation; COPD, chronic obstructive pulmonary disease.

The IVs for each phenotype investigated in this study consisted of 14–76 SNPs, explaining exposure variance ranging from 1.61% to 55.40%. The F-statistics for individual IVs (see Supplementary Table S2) and the overall F-statistics (Table 1) were all greater than 10, indicating minimal bias due to weak instrument variation. In summary, after excluding potentially pleiotropic SNPs, MR results suggested a potential bidirectional causal relationship between GERD and COPD, with COPD also increasing the risk of IBS and CP. These findings were consistent in the validation analyses. Additionally, limited evidence of a bidirectional causal relationship between PUD and COPD was found, as only positive results were present in the discovery phase. The evidence provided also indicated no causal associations between CD, UC, FD, NGE, and COPD. Figures 2, 3 provide an overview of the causal estimates and sensitivity analyses. Table 2 presents the results of heterogeneity and pleiotropy analyses for all MR analyses in this study. Supplementary Table S3 provides a more detailed breakdown of the results for all MR analyses in this study. Supplementary Figures S1–S3 present leave-one-out results for MR analyses with gastrointestinal diseases as exposures, COPD as an exposure, and validation MR analyses, respectively.

**TABLE 2:** Heterogeneity and pleiotropy of MR analysis.

Combination	Group	Q	*p*-value of Q	I2 (%)	PRESSO-RSSobs	P-RSSobs	Egger-intercept	P-Egger
GERD-COPD	Discovery	70.65	0.0014	97.22	74.19	0.003	0.0147	0.4338
	Validation	66.56	0.0028	97.22	70.31	0.004	0.0259	0.1271
PUD-COPD	Discovery	38.89	0.2988	96.14	40.98	0.337	0.0061	0.5140
	Validation	50.37	0.0270	96.17	53.87	0.033	0.0001	0.9920
IBS-COPD	Discovery	85.70	0.0248	95.50	88.69	0.015	−0.0018	0.7999
CD-COPD	Discovery	87.11	0.0007	98.86	89.81	<0.001	−0.0046	0.5250
UC-COPD	Discovery	71.68	0.0021	98.63	76.64	0.002	−0.0131	0.1108
FD-COPD	Discovery	9.34	0.7471	95.58	10.63	0.773	0.0049	0.5759
NGE-COPD	Discovery	28.38	0.0565	95.58	31.51	0.055	0.0157	0.1664
CP-COPD	Discovery	34.05	0.0258	95.68	37.86	0.020	0.0055	0.6323
COPD-GERD	Discovery	95.86	8.26E-09	95.72	102.25	<0.001	0.0103	0.1911
	Validation	71.64	2.92E-05	95.88	76.88	<0.001	−0.0073	0.3733
COPD-PUD	Discovery	66.27	0.0880	95.44	68.57	0.118	−0.0036	0.5478
	Validation	74.43	0.3060	95.96	76.64	0.318	−0.0002	0.9753
COPD-IBS	Discovery	59.89	0.4433	95.53	63.35	0.434	0.0052	0.0676
	Validation	143.13	2.57E-06	95.94	147.18	<0.001	−0.0052	0.2249
COPD-CD	Discovery	78.17	0.0174	95.20	80.60	0.020	−0.0095	0.2375
COPD-UC	Discovery	197.05	3.71E-18	95.20	204.31	<0.001	−0.0056	0.6556
COPD-FD	Discovery	71.60	0.0924	95.47	73.99	0.097	−0.0004	0.9516
COPD-NGE	Discovery	53.32	0.5771	95.37	55.42	0.590	0.0009	0.8888
COPD-CP	Discovery	73.96	0.1061	95.51	76.30	0.126	0.0023	0.6794
	Validation	68.29	0.6950	95.93	70.11	0.697	−0.0031	0.5377

Q, Cochrane’s Q; I2, I squared; PRESSO-RSSobs, RSSobs, of Global Test in MR-PRESSO; P-RSSobs, *p*-value of Global Test in MR-PRESSO; Egger-intercept, intercept of MR-Egger; P-Egger, *p*-value of Egger-intercept; GERD, gastroesophageal reflux disease; PUD, peptic ulcer disease; IBS, irritable bowel syndrome; CD, Crohn’s disease; UC, ulcerative colitis; FD, functional dyspepsia; NGE, noninfectious gastroenteritis; CP, constipation; COPD, chronic obstructive pulmonary disease.

In the discovery phase, IVW results showed that GERD increases the risk of COPD (OR = 2.005, 95% CI: 1.691–2.377, *p* = 1.21E-15), and COPD also increases the risk of GERD (OR = 1.124, 95% CI: 1.063–1.189, *p* = 4.31E-05). These results were supported by the Weighted Median method, MR-PRESSO method, and MR-RAPS method. The MR-Egger method provided causal estimates in the same direction as the IVW method, although the results were not statistically significant. In bidirectional analyses, some heterogeneity was detected by the Q-statistic and MR-PRESSO, but an I^2^ value greater than 90% suggests robust results. Additionally, the Egger intercept did not significantly differ from 0 statistically, indicating that the presence of heterogeneity did not introduce significant pleiotropic bias into the MR results. Leave-one-out analysis did not reveal any SNP that significantly influenced the causal estimates.

In the validation MR analysis, IVW results still indicated a bidirectional causal relationship between GERD and COPD (GERD-COPD, OR = 1.474, 95% CI: 1.253–1.732, *p* = 2.63E-06; COPD-GERD, OR = 1.078, 95% CI: 1.027–1.132, *p* = 0.0025). Some heterogeneity was detected by the Q-statistic and MR-PRESSO, but the Egger intercept test did not detect significant levels of pleiotropy, suggesting that heterogeneity did not introduce significant pleiotropic bias into the MR results. Leave-one-out analysis also did not identify any SNP that significantly affected the causal estimates. Therefore, the analysis results indicating a bidirectional causal relationship between GERD and COPD are considered robust.

In the discovery phase, IVW results also indicated a bidirectional causal relationship between PUD and COPD (PUD-COPD, OR = 1.165, 95% CI: 1.082–1.255, *p* = 5.55E-5; COPD-PUD, OR = 1.137, 95% CI: 1.070–1.207, *p* = 2.96E-05). The IVW estimates for PUD-COPD were supported by the MR-PRESSO and MR-RAPS methods, while MR-Egger and Weighted-Median methods did not yield statistically significant results but maintained consistency in the causal direction with IVW. The IVW estimates for COPD-PUD were supported by MR-PRESSO, Weighted-Median, and MR-RAPS, and MR-Egger also showed consistency in the causal direction with IVW. In bidirectional MR analysis, neither the Q-statistic nor MR-PRESSO detected significant heterogeneity, and an I^2^ value greater than 90% suggests robust results. The Egger intercept for MR-Egger did not significantly differ from 0 statistically, indicating no significant horizontal pleiotropy. Leave-one-out analysis also did not identify any SNPs that significantly influenced the causal estimates. However, in the validation MR analysis, no evidence was found for a bidirectional causal relationship between PUD and COPD, even though the direction of the causal estimate remained consistent with the discovery phase.

In the causal analysis between IBS and COPD, no evidence was found for IBS altering the risk of COPD. However, evidence was found for COPD increasing the risk of IBS (IVW: OR = 1.042, 95% CI: 1.012–1.073, *p* = 0.0064). The IVW results were supported by MR-PRESSO and MR-RAPS, and MR-Egger and Weighted-Median methods maintained consistency in the causal estimate direction with IVW. Neither the Q-statistic nor MR-PRESSO detected significant heterogeneity, and an I^2^ value greater than 90% suggests robust results. The Egger intercept for MR-Egger did not significantly differ from 0 statistically, indicating no significant horizontal pleiotropy. Leave-one-out analysis did not identify any SNPs that significantly influenced the causal estimates. Causal estimates were also validated in the validation phase (IVW: OR = 1.054, 95% CI: 1.012–1.098, *p* = 0.0116). This suggests that the causal estimates of COPD on IBS are relatively robust.

In the causal analysis between CP and COPD, no evidence was found for CP altering the risk of COPD. However, evidence was found for COPD increasing the risk of CP (IVW: OR = 1.128, 95% CI: 1.066–1.193, *p* = 3.22E-05). These results were supported by Weighted-Median, MR-PRESSO, and MR-RAPS, and MR-Egger method maintained consistency in the causal estimate direction with IVW. Neither the Q-statistic nor MR-PRESSO detected significant heterogeneity, and an I^2^ value greater than 90% suggests robust results. The Egger intercept for MR-Egger did not significantly differ from 0 statistically, indicating no significant horizontal pleiotropy. Leave-one-out analysis did not identify any SNPs that significantly influenced the causal estimates. Causal estimates were also validated in the validation phase (IVW: OR = 1.074, 95% CI: 1.023–1.127, *p* = 0.0039). This suggests that the causal estimates of COPD on CP are relatively robust.

For CD-COPD, UC-COPD, FD-COPD, NGE-COPD, COPD-CD, COPD-UC, COPD-FD, and COPD-NGE causal analysis, no positive results were found. Some analyses in these subgroups detected some heterogeneity, but MR-Egger intercept tests did not observe significant horizontal pleiotropy. Leave-one-out analysis did not identify any SNPs that significantly influenced the causal estimates. Therefore, it can be preliminarily concluded that there is no causal association between COPD and CD, UC, FD, and NGE.

## Discussion

The causal relationship between gastrointestinal diseases and COPD has long been a subject of uncertainty. We conducted a bidirectional two-sample Mendelian randomization (MR) study to investigate the causal relationships between eight gastrointestinal diseases and COPD. Our results indicate a bidirectional causal relationship between GERD and COPD. COPD increases the risk of IBS and CP, but IBS and CP do not alter the risk of COPD. Limited evidence suggests a bidirectional causal relationship between PUD and COPD. Furthermore, there is no evidence of causality between CD, UC, FD, NGE, and COPD. MR results are less susceptible to confounding factors and reverse causality, which may help us better understand the causal relationship between gastrointestinal diseases and COPD.

Previous observational studies have suggested an association between GERD and COPD, but making causal inferences has been challenging due to confounding factors and reverse causality. A nationwide cross-sectional study involving 141,057 COPD patients reported a GERD prevalence of 28% among COPD patients (Kim et al., 2013). A cohort study that utilized high-resolution manometry and esophageal pH monitoring with follow-up showed a correlation between the severity of GERD and the frequency of exacerbations in COPD (Bigatao et al., 2018). A meta-analysis involving 13,245 patients suggested an increased risk of COPD exacerbations associated with GERD (OR: 5.37; 95% CI: 2.71–10.64, *p* < 0.00001) (Huang et al., 2020). However, the conclusions of these observational studies are not robust, as they are subject to various confounding factors. First, smoking is a common risk factor for both GERD and COPD (Labaki and Rosenberg, 2020; Zheng et al., 2021), which is challenging to account for in observational studies. Second, COPD and asthma have significant overlap (Leung and Sin, 2017), and asthma has been causally linked to GERD (Freuer et al., 2022; Ahn et al., 2023). Third, a considerable proportion of GERD patients do not have typical reflux symptoms, but microaspiration or silent aspiration is still suspected to contribute to the development of COPD (Lee et al., 2020), making these patients easily overlooked in observational studies. The MR method we employed partially overcomes confounding factors such as smoking and asthma by excluding SNPs associated with smoking and asthma when selecting instrumental variables for GERD and COPD. We provide evidence supporting GERD as a risk factor for COPD, which is consistent with a similar MR study conducted by Cheng et al. (2023). What sets our study apart from Cheng et al.'s findings is that we also provide evidence of a positive causal effect of COPD on GERD.

Several potential mechanisms may explain the increased risk of COPD associated with GERD. First, GERD reflux can lead to chemical or aspirational pneumonia (Sanchez et al., 2020), which can further promote the occurrence and progression of COPD (Hu et al., 2015). Studies have found that controlling GERD with proton pump inhibitors significantly reduces the risk of exacerbations in COPD (Kang et al., 2023). Second, the aspiration of gastric contents can introduce irritants into the airways, particularly gastric enzymes, which can exacerbate pulmonary inflammation by damaging lung tissues, including the bronchial wall (Iliaz et al., 2016; Iov et al., 2022). Third, the immune crosstalk related to the airway microbiome is also noteworthy. Many gut microbiota can be aspirated into the lungs with reflux, which may mediate airway inflammation in COPD patients (Kayongo et al., 2022). A typical example is the ectopic colonization of *Helicobacter pylori*, and a meta-analysis has confirmed a positive correlation between *H. pylori* infection and COPD (Wang et al., 2015). *H. pylori* exotoxins have been detected in the lungs of COPD patients, which can induce the production of interleukin-8 and interleukin-6 in human lung cells (Nakashima et al., 2015).

For the mechanism by which COPD increases the risk of GERD, several factors may explain it. First, COPD patients often experience changes in intrathoracic pressure during the breathing process, especially during exhalation. When patients exhale with more force to overcome airway narrowing, it may lead to an increase in intrathoracic pressure and secondary elevation of intra-abdominal pressure (Mancopes et al., 2020). This pressure increase may increase the risk of dysfunction of the lower esophageal sphincter, thereby increasing the risk of food and gastric acid reflux into the esophagus (Siboni et al., 2022). Second, the development of barrel chest in the middle and late stages of COPD can compress the lower esophageal sphincter, causing difficulty in closure at the junction between the lower esophagus and the stomach and leading to esophageal motility disorders, thereby promoting esophageal reflux (Mittal and Vaezi, 2020). Third, COPD patients often need to use inhaled corticosteroids and other medications to manage their condition. Some drugs, especially corticosteroids and nonsteroidal anti-inflammatory drugs, may increase the risk of GERD because they can weaken the function of the esophageal sphincter (Mungan and Pinarbasi Simsek, 2017). Furthermore, systemic hypoxemia associated with COPD can exacerbate inflammation, malnutrition, and vascular dysgenesis, leading to the development of gastroesophageal dysfunction (Houghton et al., 2016).

PUD and COPD have also been considered to be related in observational studies. Many studies suggest that COPD may increase the risk of recurrent PUD, while others suggest that PUD could decrease the FEV1/FVC ratio and potentially serve as a risk factor for COPD (Siva et al., 2013). In another cohort study, after controlling for potential confounders, COPD patients had a significantly increased risk of PUD bleeding (HR = 1.93, 95% CI: 1.73–2.17, *p* < 0.001) (Huang et al., 2012). However, similar to the relationship between GERD and COPD, there is no appropriate randomized controlled trial (RCT) to establish the actual causal relationship between PUD and COPD due to interference from various confounding factors. Our MR study provides limited evidence of a bidirectional causal relationship between PUD and COPD, as the positive results were not validated in the validation set.

We believe that several reasons may have contributed to this result: our selection of instrumental variables for PUD and COPD using a *p*-value threshold of *p* < 1.0E-05 may not completely eliminate weak instrument bias, although we employed a range of sensitivity analysis methods to enhance the robustness of the results; the statistical power of the validation set we used may have been insufficient, as its total sample size was much smaller than the discovery set; there were substantial differences in population ancestry between the discovery set and the validation set. We lean towards the explanation that the statistical power of the validation set was insufficient, as our discovery analysis was conducted rigorously and did not observe significant heterogeneity and pleiotropy.

Despite the limited evidence, we still consider several possible mechanisms for the bidirectional causal relationship between PUD and COPD. Overall, both PUD and COPD are characterized by chronic inflammation. Therefore, some researchers have proposed a hypothesis that excessive pro-inflammatory mediators produced in the lungs of COPD patients may reach the intestines through the circulatory system, leading to gastrointestinal diseases; conversely, persistent gastrointestinal inflammation can exacerbate COPD due to systemic circulation (Wang et al., 2023). Regarding the increased risk of COPD associated with PUD, long-term PUD-induced inflammatory responses generate a large number of inflammatory factors, including TNF-α, IL-1, IL-6, IL-8, etc. (Fagundes et al., 2021). They can reach the lungs through the bloodstream and lymphatic system, promoting the occurrence and development of pulmonary inflammation and mutually reinforcing each other in this process (Fagundes et al., 2021). Oxidative stress induced by PUD can also exacerbate COPD inflammation (Perez et al., 2017). Malnutrition or even malnutrition in PUD patients can reduce the body’s ability to resist inflammation-induced lung damage (Calder et al., 2018). Conversely, the gastrointestinal mucosa is highly reactive to hypoxia, oxidative stress, and other factors (Sverden et al., 2019), and the chronic systemic hypoxia, oxidative stress, and high inflammatory state caused by COPD become important causes of PUD (Ferrera et al., 2021).

It is worth noting that although our results suggest evidence of bidirectional causality between GERD, PUD, and COPD, it does not necessarily imply a causal relationship. This is due to the inherent limitations of Mendelian randomization and the specific limitations of our study. Despite employing various methods to mitigate potential confounding factors, we cannot completely rule out the possibility that GERD, PUD, and COPD share some genetic factors that could be influenced by common underlying factors.

Previous observational studies have indicated a certain correlation between IBS and COPD (Lai et al., 2020; Chiu et al., 2022), but this association has not been clearly established. Our MR study suggests that COPD increases the risk of IBS, while IBS does not alter the risk of COPD. There are several potential mechanisms that may explain this causal relationship. First, the long-term systemic chronic inflammation caused by COPD can disrupt intestinal immunity and the gut microbiome environment (Bowerman et al., 2020), further promoting IBS (Grayson, 2016; Canakis et al., 2020). The chronic systemic hypoxia resulting from COPD can lead to increased intestinal epithelial cell permeability and dysbiosis (Sprooten et al., 2018), further increasing the risk of IBS (Zhang et al., 2022). COPD patients often experience comorbid depression and anxiety (Pumar et al., 2014), which can be detrimental to IBS patients (Fond et al., 2014).

The association between CP and COPD has received less attention in previous research, but some studies have observed an increased risk of CP in COPD patients (Sun et al., 2013; Gursoy Coskun et al., 2021). Our MR results are consistent with observational study findings, indicating that COPD increases the risk of CP, but CP does not increase the risk of COPD. There are several possible mechanisms for this association. Firstly, COPD patients often experience symptoms such as shortness of breath and respiratory distress, which may lead to reduced physical activity levels. Insufficient physical activity can affect normal bowel motility and defecation rhythms, potentially triggering CP (Booth et al., 2012). Secondly, COPD is a chronic inflammatory disease, which may affect intestinal function, including intestinal motility, thereby increasing the risk of CP (Shatri et al., 2023). Additionally, gut microbiome dysbiosis and emotional changes induced by COPD may also contribute to an increased risk of CP (Bowerman et al., 2020; Shatri et al., 2023).

The relationship between CD, UC, FD, NGE, and COPD has also garnered attention in previous research, and observational studies have suggested a strong association between CD, UC, FD, NGE, and COPD (Brassard et al., 2015; Chiu et al., 2022; Mathews et al., 2022). However, our MR study did not find evidence of an association between them. It is important to note that the absence of evidence in our MR study does not definitively rule out the possibility of causal relationships between CD, UC, FD, NGE, and COPD. Larger MR studies or randomized controlled trials may be necessary to further investigate these potential associations.

Compared to previous observational studies, our MR study has several advantages. It is less susceptible to confounding factors and reverse causality. Each summary data set had a relatively large sample size, providing sufficient statistical power. Confirmatory MR analysis and extensive sensitivity analyses assessed pleiotropy and potential statistical bias, enhancing the robustness of the results.

However, this study also has notable limitations. MR studies can be affected by horizontal pleiotropy, and we employed various methods to mitigate this bias. The population data selected for our study were predominantly of European ancestry, which limits the generalizability of causal relationships to other populations. Negative results in MR studies do not completely rule out causal associations because genetically determined exposures may not fully represent true exposures, and stricter selection of instrumental variables may result in negative results. Finally, due to the lack of appropriate data, we were unable to conduct gender-stratified and population-stratified analyses.

## Conclusion

In summary, our results support a bidirectional causal relationship between GERD and COPD, while COPD increases the risks of IBS and CP. Additionally, our study also suggests a bidirectional causal relationship between PUD and COPD, although the evidence strength may be insufficient and further research is needed to confirm this. Furthermore, we provide evidence that there is no causal association between CD, UC, FD, NGE, and COPD. Physicians and patients should pay greater attention to the management of gastrointestinal diseases and COPD patients, as the relationship between them may not be simply susceptibility to the environment. Further exploration of the potential mechanisms of mutual interference between GERD, PUD, and COPD, as well as the mechanisms by which COPD alters the risks of IBS and CP, is warranted.

## Data Availability

The datasets presented in this study can be found in online repositories. The names of the repository/repositories and accession number(s) can be found below: The summary data for gastroesophageal reflux disease is available at “https://gwas.mrcieu.ac.uk/”; the summary data for peptic ulcer disease is available at “https://cnsgenomics.com/content/data”; the summary for Irritable bowel syndrome, Crohn’s disease, ulcerative colitis, non-infectious gastroenteritis and constipation data are available at “https://www.ebi.ac.uk/gwas/”; data for chronic obstructive pulmonary disease (discovery) is available at “https://www.ebi.ac.uk/gwas/”; data for chronic obstructive pulmonary disease (discovery) and constipation are available at “https://www.finngen.fi/en” to obtain.

## Abbreviation

COPD, chronic obstructive pulmonary disease; GWAS, Genome-wide association study; MR, Mendelian randomization; GERD, gastroesophageal reflux disease; PUD, peptic ulcer disease; IBS, irritable bowel syndrome; CD, Crohn’s disease; UC, ulcerative colitis; FD, functional dyspepsia; NGE, non-infectious gastroenteritis; CP, constipation; FEV1, forced expiratory volume in 1 second; FVC, forced vital capacity; SNP, single nucleotide polymorphism; OR, odds ratio; IVW, inverse variance weighting; MR-Egger, MR-Egger regression; MR-PRESSO, Mendelian Randomization Pleiotropy RESidual Sum and Outlier (MR-PRESSO) test; MR-RAPS, Mendelian Randomization Robust Adjusted Profile Score.
