# Supplementary figures and images for: Common gastrointestinal diseases and chronic obstructive pulmonary disease risk: a bidirectional Mendelian randomization analysis
Source: Front Genet. 2023 Nov 16;14:1256833. doi: 10.3389/fgene.2023.1256833 (PMC10690629; doi:10.3389/fgene.2023.1256833)

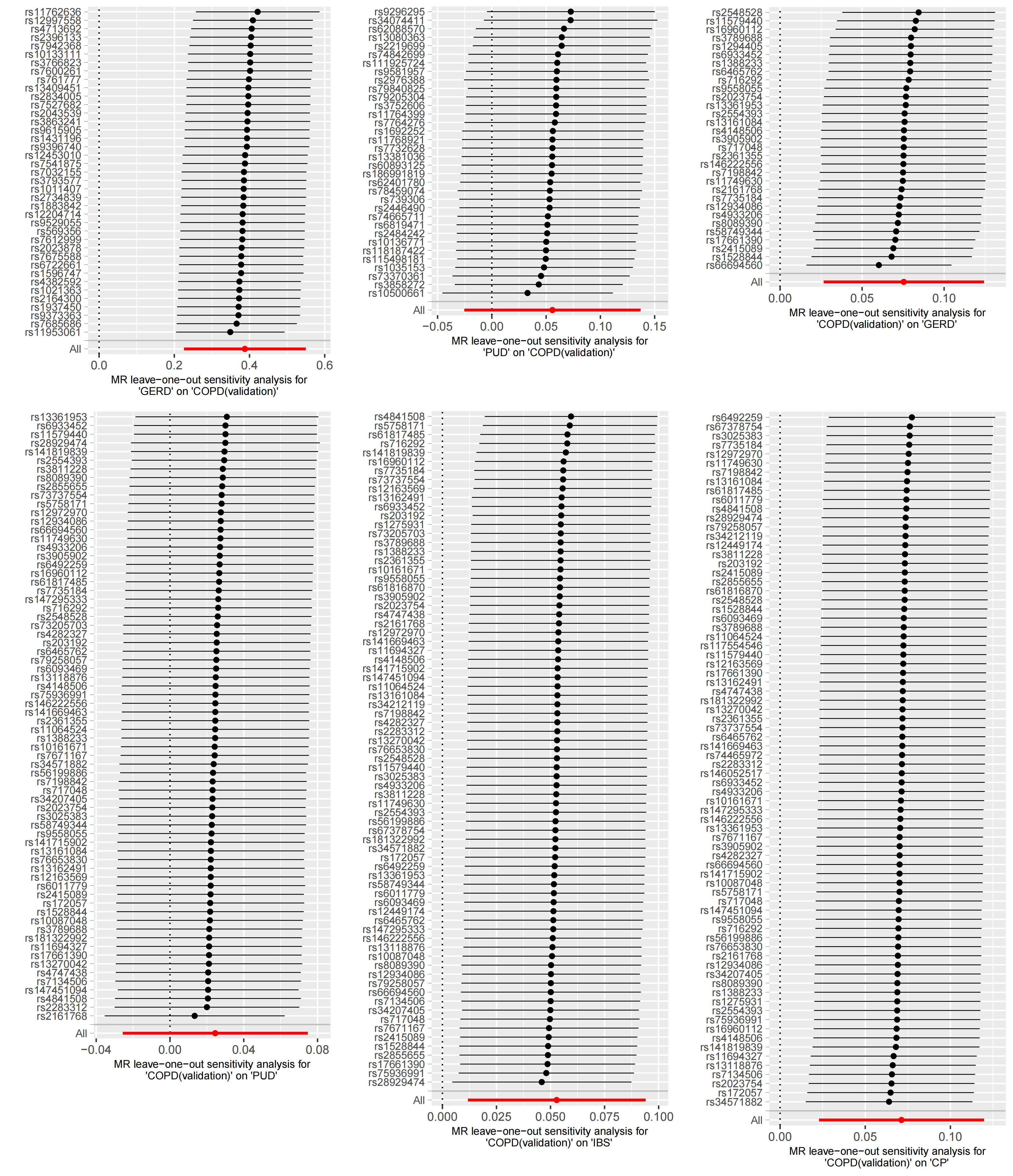

Supplement: Supplementary file 2 [file Image3.jpg]

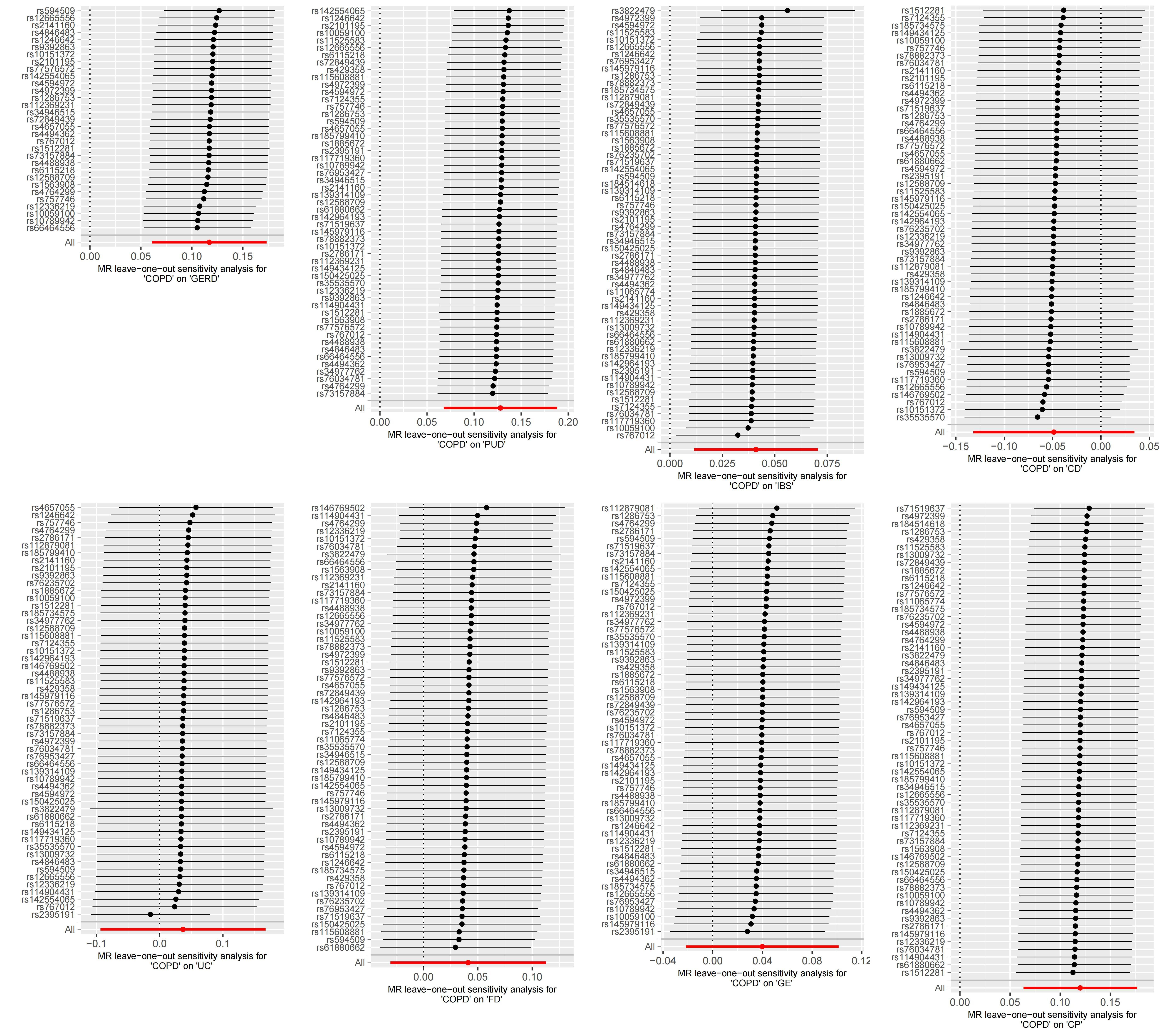

Supplement: Supplementary file 3 [file Image2.jpg]

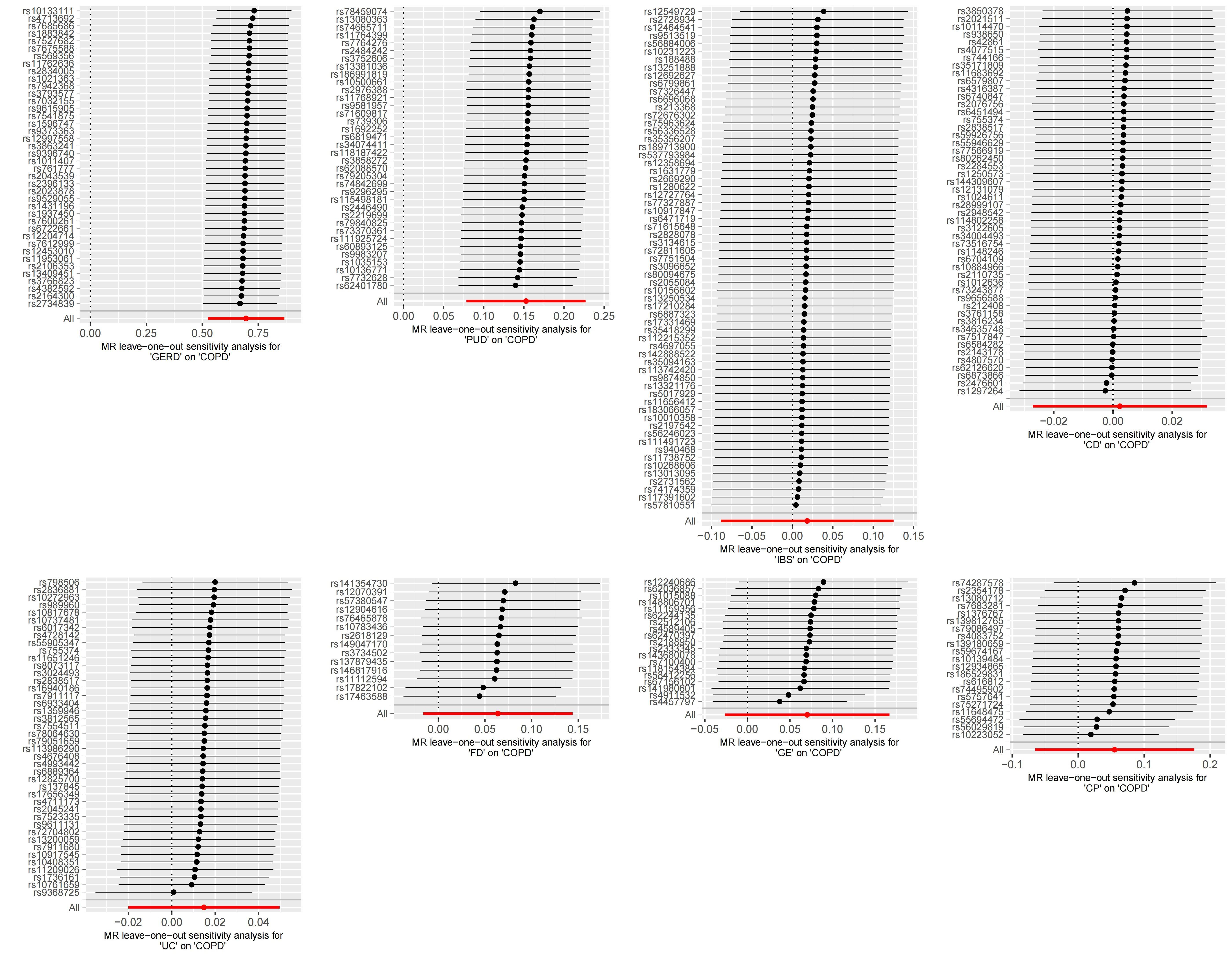

Supplement: Supplementary file 4 [file Image1.jpg]
